# Eye-Tracking for Clinical Ophthalmology with Virtual Reality (VR): A Case Study of the HTC Vive Pro Eye’s Usability

**DOI:** 10.3390/healthcare9020180

**Published:** 2021-02-09

**Authors:** Alexandra Sipatchin, Siegfried Wahl, Katharina Rifai

**Affiliations:** 1 Institute for Ophthalmic Research, University of Tübingen, 72076 Tübingen, Germany; siegfried.wahl@uni-tuebingen.de (S.W.); katharina.rifai@medizin.uni-tuebingen.de (K.R.); 2 Carl Zeiss Vision International GmbH, 73430 Aalen, Germany

**Keywords:** eye-tracking, head-mounted display (HMD), virtual reality (VR), ophthalmology, usability methods

## Abstract

Background: A case study is proposed to empirically test and discuss the eye-tracking status-quo hardware capabilities and limitations of an off-the-shelf virtual reality (VR) headset with embedded eye-tracking for at-home ready-to-go online usability in ophthalmology applications. Methods: The eye-tracking status-quo data quality of the HTC Vive Pro Eye is investigated with novel testing specific to objective online VR perimetry. Testing was done across a wide visual field of the head-mounted-display’s (HMD) screen and in two different moving conditions. A new automatic and low-cost Raspberry Pi system is introduced for VR temporal precision testing for assessing the usability of the HTC Vive Pro Eye as an online assistance tool for visual loss. Results: The target position on the screen and head movement evidenced limitations of the eye-tracker capabilities as a perimetry assessment tool. Temporal precision testing showed the system’s latency of 58.1 milliseconds (ms), evidencing its good potential usage as a ready-to-go online assistance tool for visual loss. Conclusions: The test of the eye-tracking data quality provides novel analysis useful for testing upcoming VR headsets with embedded eye-tracking and opens discussion regarding expanding future introduction of these HMDs into patients’ homes for low-vision clinical usability.

## 1. Introduction

Eye-tracking in virtual reality (VR) for ophthalmology practices is a promising emerging field for objective and at-home diagnostic and treatment purposes. Online analysis of eye-tracking data is currently being used in a VR environment for hands-free perimetry testing [1,2,3] and dynamic VR visual enhancements [4,5].

Online gaze tracking for virtual reality perimetry implements an objective, mobile and portable perimetry where the gaze replaces the patient’s response. A perimetry test is usually used to identify the amount of visual loss in the central and peripheral visual field. For example, the standard perimetry test, the Humphrey visual field analyzer (HFA, Carl Zeiss Meditec Inc., Dublin, CA, USA) is used to test a specific point in the visual field. Subjects are asked to press a button whenever they see a light target on a 2D plane extending ±30° temporally and nasally. The concept of virtual perimetry [6] has shown increasing potential with multiple studies testing its comparability to the standard HFA [7,8,9,10,11]. Virtual reality perimetry introduces a visual grasp mode, based on eye movements instead of subjective button presses to collect the patient responses. It has the advantage of overcoming long periods of fixation of peripheral stimuli common to standard perimetry [12]. In a visual-grasp modality, eye-tracking data automatically identifies the responses. During central fixation, a stimulus appears at a new fixation area, and it induces an automatic gaze reflex change towards the new target. When the gaze change is consistent with the change in the target position, the test identifies that part of the visual field as being intact. Testing the visual field in a VR environment expands perimetry in the direction of a mobile application that can easily be introduced into the patient’s home’s comfort.

Virtual reality technology has the additional potential of overcoming the limits of conventional rehabilitative therapy regarding its actual usage in at-home settings thanks to the engaging nature of VR [13]. New up-coming extended reality assistive software techniques have started to use eye-tracking to offer a more reactive user interface. These studies take the gaze input to enhance low vision via a display that continuously and dynamically updates where the subject is looking. The eye-tracking application’s main advantage is that it can be customized to each patient’s needs with the required enhancement being applied to the damaged visual field uniquely in all of its parts. [4].

For these applications, the eye-tracking data quality has to be accurate, precise, and temporally exact for effective usability. For an objective visual grasp modality in VR, accuracy and precision of fixation are essential for the correct assessment of visual field loss. For visual enhancement usage, eye-tracking temporal precision is important: the actual timing between a shift in the eye-tracking data and a relative change in the VR headset screen should ideally last less than 54 milliseconds (ms), a saccade duration [14,15,16,17,18,19,20], so that the applied enhancement remains undetected and a comfortable user interface is maintained. Indeed, this type of study assumes that the participant is not aware of the changing display while performing eye movements such as saccades, since, during a saccade, the stimulus is not perceived [21,22].

The current limitation is that eye-tracking quality varies by software, hardware, and manufacturer. Currently, low vision clinical studies, using virtual reality, have used different hardware and reported information from the manufacturer to indicate eye-tracking hardware capability and usability in their studies [1,2,3,4,5]. No study until now has questioned the reliability and suitability of the available manufacturers’ information for online low-vision applications.

For engaging at-home treatments, an empirical assessment of the status-quo of the eye-tracking hardware is needed, so that reliable information can be used for assessing the ready-to-go potentiality of VR headsets with an embedded eye-tracker. Accordingly, the first case study that investigates the status-quo of a commercial head-mounted-display (HMD) with embedded eye-tracking is being proposed. The status-quo of the HTC Vive Pro Eye is tested. The results obtained will provide better guidance for future research using this hardware for clinical studies. The current pilot study describes two new types of methodology to test eye-tracking data quality for low-vision use. For VR online perimetry testing, eye-tracking data quality is investigated at large visual fields up to ±30°, and the influence of different screen regions of the VR headset over data quality is tested. For mobile applications, the current study also tests for data quality during movement. For VR online visual enhancement applications, a Rasberry Pi system, non-expensive, and with an automatic method for temporal precision calculation, is used. These eye-tracking testing tools are essential for future investigation of upcoming and more advanced commercial virtual reality headsets with embedded eye-tracking such as StarVR One (Starbreeze Studios AB, Stockholm, Sweden) and Pico Neo 2 Eye (Pico Interactive, San Francisco, CA, USA) intended for online low-vision assessment.

## 2. Materials and Methods

### 2.1. Study Group

Eleven participants took part in the data quality assessment test (six females and five males, mean age 28.73, standard deviation (SD): ±2.49 years) with 11 participating for the head-still and 10 being re-tested for the head-free condition. The direct end-to-end method for latency required no participants.

### 2.2. Set-Up

For the virtual experiment, the Unity 2019.1.10f1 version was used as a design tool, with C# as a programming language, running on a computer with Windows 10 Home, having a 64-bit operating system, an Intel Core i7 -7700HQ, 2.8 GHz, 16 GB RAM, and an NVIDIA GeForce GTX 1070 GDDR5 graphics card. A single-board computer was used, the Raspberry Pi (Raspberry Pi Foundation, Cambridge, England, UK), model B 2018 [23], controlling a Raspberry Pi camera (Version 2.1 [24] with a capability of 120 Hz,) for the end-to-end direct latency tests. Eye-tracking data was collected in a virtual environment using the HTC Vive Pro Eye [25] with built-in Tobii eye tracker (Core SW 2.16.4.67) with an accuracy estimation of 0.5°–1.1° and a sampling frequency of 120 Hz (HTC Corporation, Taoyuan, Taiwan). Tobii Pro SDK v1.7.1.1081 [26] (Tobii Technology, Stockholm, Sweden) and Vive SRanipal SDK v1.1.0.1 [27] (HTC Corporation, Taoyuan, Taiwan) are used to access non-filtered and filtered eye-tracking data, respectively. The embedded HMD’s calibration system is used to calibrate eye-tracking data for each participant.

The HTC Vive headset contains two active-matrix organic light-emitting diode (AMOLED) screens, with a resolution of 2.880 × 1.600 pixels in total with a refresh rate of 90 Hz and a field of view of 110°.

### 2.3. Study Parameters

#### 2.3.1. Eye-Tracking Accuracy and Precision Measurements: Head-Still and Head-Free Tests

**Figure 1:**
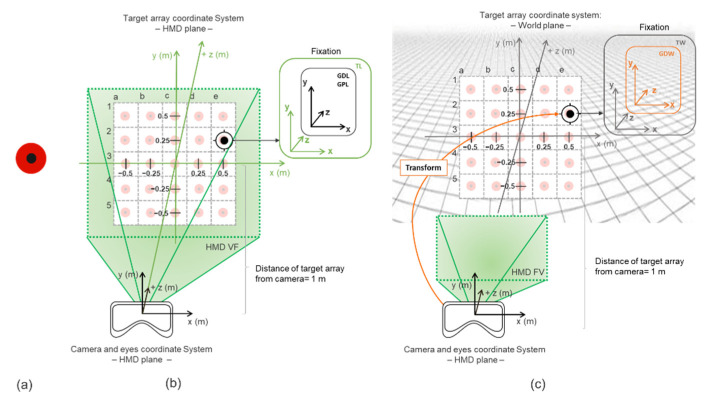
The target (**a**) is a virtual object with two concentric circles. In the head-still condition (**b**) the targets are locally fixed to the head-mounted display (HMD) (referred in the figure as TL), the same as for gaze direction (GDL in the figure) and gaze position (or GPL). In the head-free condition (**c**), precision is tested on the 3D world plane. Fixation is re-referenced (transform), so that target (as in TW) and gaze direction (or GDW) are on a world plane.

Accuracy is the average angular error between the measured and the actual location of the intended fixation target. Precision is the spread of the gaze positions when a user is fixating on a known location in space [20,28]. Accuracy and precision were tested in a virtual environment where fixation targets (Figure 1a) were two concentric circles, one internal black and one external red circle, with a radius of 0.72 degrees of visual angle, positioned at 1 m in a Unity world coordinate system. To avoid alteration of eye-tracking samples, the Tobii Pro SDK was used to access non-filtered data, and the luminance of the targets was kept constant in the virtual environment to avoid pupil dilatation due to changes in stimulus brightness, which is known to affect eye-tracking data [28,29,30]. Two separate conditions were investigated: head-still and head-free. Subjects performed the task in both conditions in a seated position on a chair.

In the head-still condition, the target position was fixed to the HMD. As a result, if the headset moved, targets would move along with it. This way, accuracy, and precision could be tested across the headset 2D plane covering a visual field of ± 26.6° (Figure 1b). The target would appear at 25 different sample positions with 5 columns and 5 rows. The target position appearance was randomized, and each target was displayed for 5 s [31] with 5 repetitions (5 sec/target × 25 targets × 5 repetitions = 625 s, approximately 10 min and a half). When a target appeared, the subject had to fixate on it until it disappeared while keeping their head still.

In the head-free condition, targets were positioned in a world-fixed coordinate system, and as a result the targets did not move with the HMD but had a fixed position on the world plane (Figure 1c). The subject had to move their head instead so that precision and data loss could be tested for head-movement effects. As for the head still condition, targets were distributed across 25 different positions at a similarly large visual field. A central fixation target was added (coordinates: [0,0,0]) at the end of each target presentation that lasted 2 s, to make the participates come back to the same referencing point (5 sec/target + 2 sec/central target × 25 targets × 5 repetitions = 875 s, approximately 15 min). During this condition, subjects had to saccade towards the appearing target, fixate on it and then move their head naturally, while fixating, towards the position where it appeared. As soon as the target could be fixated centrally, subjects were instructed to keep the head stable until the target disappeared.

#### 2.3.2. Eye-Tracking Temporal Precision Measurements: Eye-Detection and Gaze-Contingent Tests

**Figure 2:**
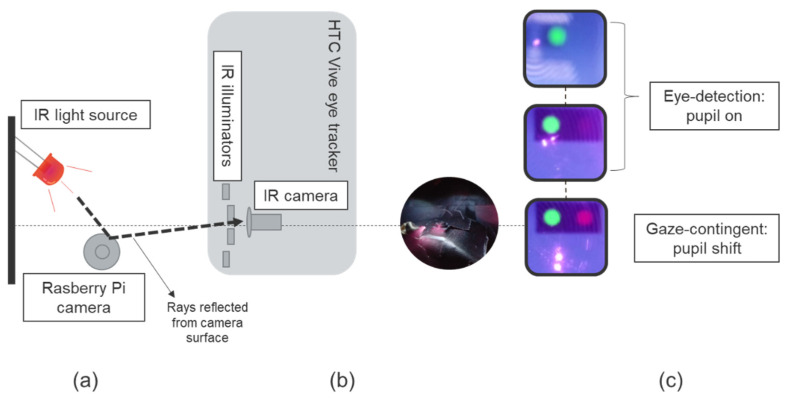
Infrared (IR) light source (orange) illuminates the Raspberry Pi camera: (**a**) schematic and (**b**) picture of the camera. The Rasberry Pi records it as a reflection on the HMD lenses (**c**), pink dot. The IR camera inside the HTC Vive Pro Eye captures the reflected rays by the Raspberry Pi camera as an artificial eye. (**c**) Recorded events: greed dot referring to pupil-on and red dot indicating pupil-shift events.

Temporal precision is the average end-to-end delay from the tracked eye’s actual movement until the recording device signals that the movement has occurred [28]. A new method is described, which uses a low-cost configuration (Figure 2a): a Raspberry Pi single-board computer that controls the output of infrared light-emitting diodes (LEDs) and records, with a Raspberry Pi camera, reflections from the camera and eye-tracking events displayed by the headset produced by these infrared reflections when the LEDs are on (Figure 2a,b). A virtual environment was used, running on a computer that displayed the VR Positioning Guide Prefab, incorporated in the Tobii Pro SDK (Figure 2c, first image) and a similar programmed version of the Prefab SRanipal SDK (Figure 2c, last two images) to display the events. Ten different videos for each SDK were recorded. The Raspberry Pi tested temporal precision using two different scenarios.

**Figure 3:**
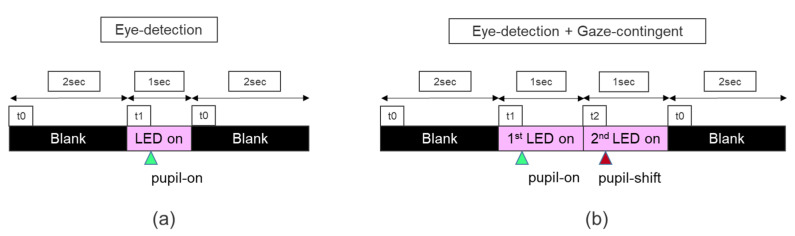
(**a**) The eye-detection scenario was tested both for the Tobii and the SRanipal SKD. The test has two-time series; at t0 the LED is off (2 s) and t1 two pairs of LEDs are turned on (1 s): pupil-on is simulated (green triangle). (**b**) For the SRanipal SDK, an eye-detection scenario is produced followed by a gaze-contingent scenario: the Raspberry system turns on the first pair of LEDs (t1) for 1 sec, then switches off the first one while turning on the second pair (t2): pupil shift is simulated (red triangle).

The eye tracker is firstly tricked into the detection of an eye: the eye-detection scenario. The Raspberry Pi turns on two infrared LEDs for 1 s, leading to a pupil-on event with the appearance of a green dot (Figure 2c, first two images and Figure 3a). Afterward, the LEDs are switched off for 2 s (Figure 3a). A total of 33 infrared LED on and 33 LED off time series are tested. This first scenario is tested both with Tobii Pro SDK and SRanipal SDK to check for differences in latency when identifying an eye appearing between two different SDKs. The second scenario is tested only with the SRanipal. Therefore, when using the Tobii Pro SDK there are 330 repetitions and a recording time of 16.7 min.

While recording with the SRanipal, a second scenario is introduced as a modified version of an artificial saccade generator [32]. Secondly, the Raspberry Pi tricks the eye-tracker into an abrupt change in gaze position of the recognized artificial pupil, i.e., the gaze-contingent scenario. For this scenario, two additional infrared LEDs were placed at a 1cm distance from the other two, and the Raspberry Pi turned them on for 1 s, at the same time as it turned off the first two that had previously been used to produce the green dot event (Figure 3b). Turning on the second pair of infrared LEDs simulated an abrupt change in the previously recognized artificial pupil’s gaze position. This change was followed by a pupil shift event with a bright red dot (Figure 2c, lower image, and Figure 3b). The pupil shift event did not disrupt the first pupil-on event since the display of this event was programmed such that a green dot would still be shown as long as an eye is being detected. For the SRanipal SDK, each scenario had 33 infrared LED on-off time series; therefore, 660 repetitions were recorded with a total time of 21.2 min.

### 2.4. Statistical Analysis

#### 2.4.1. Pre-Processing: Eye-Tracking Accuracy and Precision

**Figure 4:**
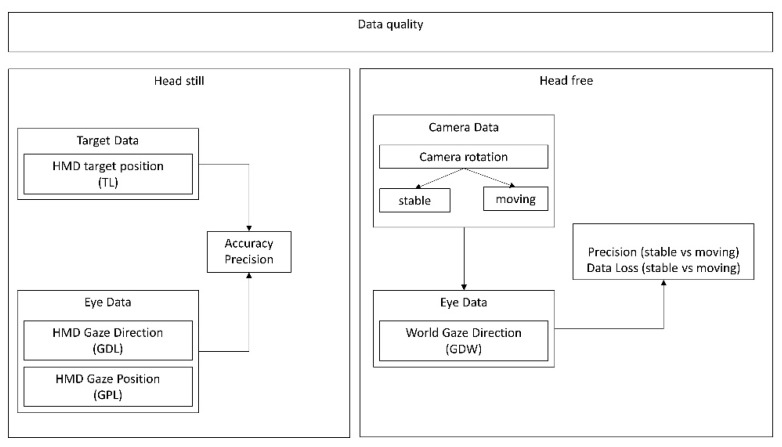
For the head-still condition, the HMD-local target position (TL), HMD-local gaze direction (GDL), and HMD-local gaze position (GPL) are used for accuracy and precision measures. In the head-free condition, camera rotation is used to separate world gaze direction, distinguishing stable head and moving head. The world gaze direction (GDW) is used to investigate precision and data loss between periods of stable fixations and periods of moving fixations.

In the head-still condition, both for the left and right eye separately, the HMD-local gaze position (vector of eye position measured in millimeters from the center of the HMD) and HMD-local gaze direction (a normalized vector referenced in HMD-local’s coordinate system pointing from the pupil towards the virtual object) were selected. Local gaze direction (Figure 4, GDL) and local position vector (Figure 4, GPL) were then calculated, with the average of both eyes’ coordinates [31]. The local target position was also saved at every sample (Figure 4, TL). For each data sample, the targets were re-referenced to the eye (TL-E) by subtracting the local eye’s position vector from the local target’s coordinates (target vector (TL)—eye position vector (GPL)).

Afterwards, the angle between the local gaze direction vector (GDL) and the local target-eye vector(TL-E) was calculated using the Formula (1) to estimate the angle between two vectors (angleV) [31].

(1) $$\mathrm{angleV}\;\left({}^{\circ}\right)\left(\mathrm{Local}\;\mathrm{coordinates}\right)=\tan^{-1}(\mathrm{norm}\left(\left(\mathrm{GDL}\times \;\mathrm{TL}-E\right),\left(\mathrm{GDL}\;\cdot \mathrm{TL}-E\right)\right))$$

Norm normalizes the vector; cross (×), and dot (·) calculate the cross and the dot product, respectively.

In the head-free condition, the world gaze (Figure 4, GDW) was selected as an already averaged vector between the left and right eye as provided by the Tobii Pro SDK Prefab. The world gaze direction provided by the Prefab is calculated as follows: the HMD-local gaze position is used to re-reference the new gaze direction [31]. In the head-free condition, for each data sample, to separate between fixation during head-non-moving (head-free_stable_) and fixation during head-moving phases (head-free_moving_), the differential of the speed of the HMD’s rotation quaternion was calculated (Figure 4, camera data), rotated around a normalized vector.

For the analysis, the first 500 ms [31] after the target appearance were discarded. That was considered as the time a subject used to direct the gaze. Gaze points where no eye could be tracked were excluded from the analysis both for the left and the right eye in both conditions. For the data loss analysis, gaze points where no eye was detected were kept.

#### 2.4.2. Eye-Tracking Accuracy and Precision

Accuracy is defined as the mean of all the angles (angleV) calculated between GDL and TL-E using the formula described in Equation (1). To calculate the eye-tracker’s spatial precision [33] the common practice was used, i.e., the root mean square (RMS) of the inter-sample angular distances between successive gaze directions.

For the head-still condition before averaging between the two eyes, a one-way ANOVA tested for differences in accuracy between the two eyes. To analyze changes in eye data quality across the population, tested percentiles were calculated. An overall average and an average for different percentiles of users for accuracy and precision were computed. A one-way ANOVA way tested how accuracy and precision differ across screen regions with the horizontal line as the independent factor and the vertical lines as levels. Differences observed across the horizontal line might be an indication of the altering of eye-tracking data quality induced by reflections from vision corrections [34]. As an additional precision indicator, a bivariate contour ellipse area (BCEA) for left, right, and the average of the two eyes was also plotted to show the area that encompasses 50% of fixation points around the mean for each given target.

In the head-free condition, the average precision as RMS between the successive GDW was calculated and a one-way ANOVA tested how precision is affected by phases of stable head and moving head while subjects fixated on the target. The data loss percentage [35] was calculated using the Formula (2):

(2) $$\mathrm{Data}\;\mathrm{loss}\;\left(\%\right)=100\times \frac{\mathrm{Nsamples}-\mathrm{Nvalid}\_\mathrm{samples}}{\mathrm{Nexpected}\_\mathrm{samples}}$$

Nsamples represents the number of data samples recorded after excluding the initial 500 ms, and Nvalid_samples are the number of samples during which a valid gaze position was recorded.

#### 2.4.3. Eye-Tracking Temporal Precision

**Figure 5:**
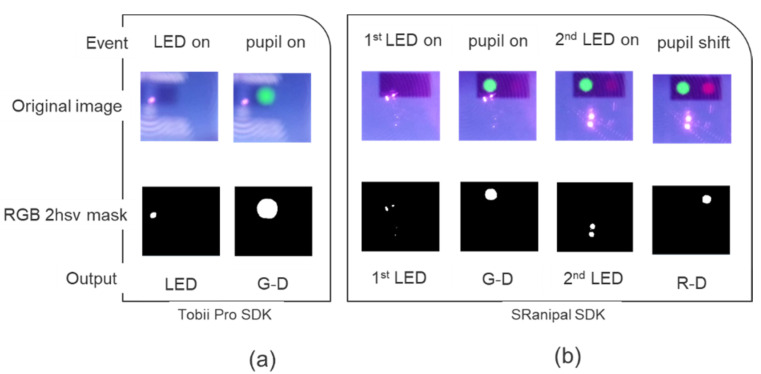
Original image: original frame pictures showing different events for (**a**) the Tobii Pro SDK (event: LED on and pupil on) and (**b**) the SRanipal SDK (event: first LED on, pupil on, second LED on, pupil shift). RGB 2hsv mask: application of Matlab’s image segmentation mask to the selected events. The masks indicate how many pixels are in each event for the LED and dot events for (**a**) the Tobii Pro SDK (output: LED, G-D) and (**b**) SRanipal SDK (output: first LED, G-D, second LED, R-D).

The recorded videos were converted into images frame-by-frame through a converter program (Free Video to JPG Converter, version 5.0.101). A new automatized method was programmed to detect the elapsed frames between the LED’s onset and the onset of the different dots. The Color Thresholder app was used from the Matlab Image Processing Toolbox (version 10.4) to manipulate sample frames’ color components via a hue, saturation, value (2HSV) color space. Three separate red-green-blue (RGB) 2HSV segmentation masks were created: one for all the LED’s reflection on the HMD (Figure 5a, LED; Figure 5b, first LED and second LED), one for the appearance of the green dot (Figure 5a,b, G-D), and one for the appearance of the bright red dot (Figure 5b, R-D). The masks indicated how many pixels in the frame contained the events; this permits automatic identification of events. When using the SRanipal, to differentiate between the first and the second pair of LEDs, for each frame, the script attributed a flag whenever the number of pixels was greater or smaller than given values. This flag is made possible since the second pair of LEDs cause a bigger reflection area, therefore a bigger number of pixels on the resulting mask (Figure 5b, second LED on and second LED mask).

For the eye-detection scenario, both when using the Tobii Pro SDK and the SRanipal SDK, the script automatically counted the number of frames between LEDs and the green dot onset. For each frame, the script attributed a flag whenever the generated LED mask or G-D mask had a number of pixels greater than 10. Additionally, when using the SRanipal, the script identified the first LED pair when the first LED mask had a number of pixels greater than 10, but also smaller than 250.

The count started with the second pair of LEDs’ onset for the gaze-contingent scenario and ended with the bright red dot appearance. When the bright red dot was on for each frame, the script attributed a flag to the corresponding R-D mask when it contained a number of pixels greater than 10. For the second pair of LEDs, on every frame the script attributed a flag, whenever the number of pixels was greater than 300, to the second LED mask.

For the analysis both for eye-detection and gaze-contingent scenarios a histogram and a boxplot were plotted with the resulting intervals between events and tested for normal distribution with a one-sample Kolmogorov-Smirnov test. Temporal precision was calculated as the median of frame numbers elapsed between the LED and the different dot event multiplied by each video frame’s mean duration.

## 3. Results

### 3.1. Pre-Processing: Eye-Tracking Accuracy and Precision

**Figure 6:**
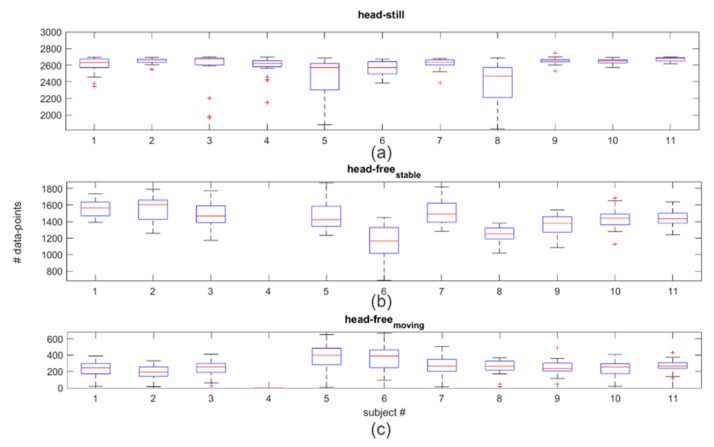
Bar plots with error bars and outliers (red plus dots) represent the median of data point for each subject for the 25 targets after data pre-processing for the head still (**a**) and head-free condition (**b**,**c**). The head-free condition is divided between stable (**b**) and moving head (**c**) periods.

After data selection for each target, subjects had a median of 2638 data points in the head-still (Figure 6a) condition. The head-free_stable_ (Figure 6b) condition had a median of 1408, and the head-free_moving_ had a median of 251 points per target (Figure 6c).

### 3.2. Eye-Tracking Accuracy and Precision

**Figure 7:**
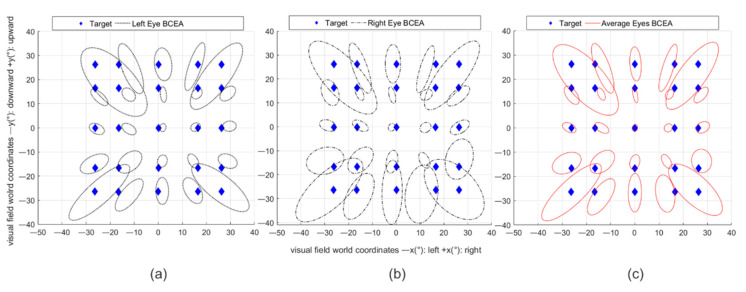
Covariance ellipses (BCEA) are fitted to the left eye’s (**a**), right eye’s (**b**), and both eyes’ (**c**) gaze points corresponding to all fixations of the same target across all subjects. The blue diamonds are the targets.

In the head-still condition, the one-way ANOVA resulted in no significant differences in accuracy between the two eyes (F (1, 20) = 0.81, *p* = 0.38; mean left eye: 4.16° SD: ±1.49 and mean right eye: 4.75° SD: ±1.63). For this reason, the average across eyes (mean average of both eyes: 4.16°, SD: ±1.40) was used for the analysis. Precision has a mean of 2.17°, SD: ±0.75. The BCEA and mean accuracy angle and precision values per target show that the accuracy and precision of the estimated gaze are worse at the outermost horizontal regions and that the central line has higher accuracy and precision than the most externally positioned targets, with the highest level of accuracy and precision for the central target (Figure 7a–c and Table 1). Comparing horizontal regions, a one-way ANOVA revealed that there is a significant difference in accuracy and precision (F (4, 50) = 3.35, *p* = 0.02 for accuracy; F (4, 50) = 3.6, *p* = 0.01 for precision). Post-hoc *t*-tests (Bonferroni corrected) show the center as being more accurate than the upper horizontal (*p* < 0.03, central row mean offset: 2.26°, SD: ±0.73; upper row mean offset: 6.16° SD: ±5.50), and as more precise than the lower horizontal (*p* < 0.01, central row RMS mean: 1.63° SD: ±0.30 and the lowest row RMS mean: 3.15°, SD: ±2.00).

**Figure 8:**
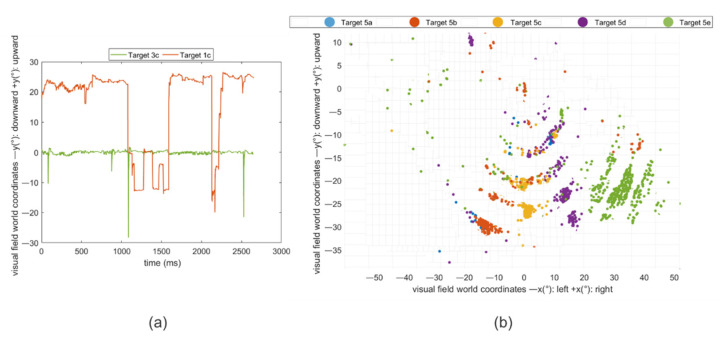
(**a**) Fixations plotted across the time for one subject evidence an unstable fixation of the upper-central target, target 3c, compared to a central one (green), target 1c. (**b**) Starting from left to right, target 5a is the most left target, positioned in the last row, and target 5e is the most right target. Blue, orange, yellow, lilac, and green are the dispersed fixation points belonging to target 5a, 5b, 5c, 5d, and 5e, respectively.

Fixational eye movements of single subjects were plotted that revealed unstable fixation patterns for the upper row (Figure 8a) and deviations for the lower (Figure 8b). Accuracy and precision become worse for different quantiles of users (Table 2). Starting from the third quartile, accuracy and precision dropped. The accuracy passed from a visual angle of 3.21° to 4.88° and 6.06°, and precision passed from 1.63° to 2.51° and 3.55° from the first quartile to the third, and the 90th percentile, respectively.

In the head-free condition, there is an overall average precision of 1.15°, SD: ±0.69. Under head-movement one-way ANOVA revealed a significant difference in precision between head-free_stable_, compared to phases of head-free_moving_ (F (1, 18) = 8.64), *p* < 0.01; RMS mean_stable_: 0.76°, SD_stable_: ±0.39, RMS mean_moving_: 1.54°, SD_moving_: ±0.74) with higher imprecision during periods in which subjects were moving their head. As to data loss, there is a double amount of data slippage in the head-free_moving_ phase compared to when subjects were not moving their head (7.56% of data spillage compared to 3.69% of data spillage).

### 3.3. Eye-Tracking Accuracy and Precision

**Figure 9:**
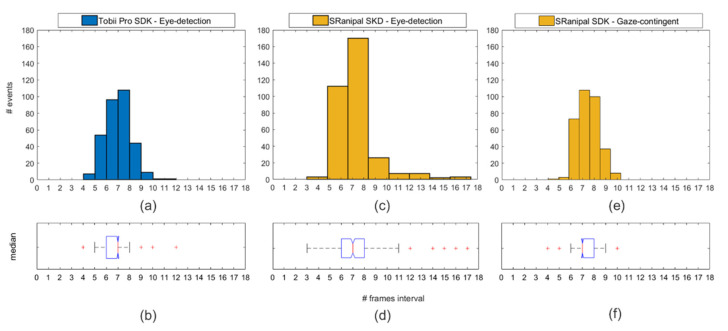
(**a**) The blue histogram indicates the number of the frame distribution when using the Tobii Pro SDK in the eye-detection scenario. (**b**) Box plot of the median inter-frame interval. (**c**) The number of the frame distribution in the eye-detection scenario. (**d**) Box-plot with the median of the eye-detection scenario. (**e**) Number of the frame distribution in the gaze-contingent scenario. (**f**) Box-plot of the median of the gaze-contingent scenario. All box-plots are plotted with error bars and outliers (red plus dots).

The one-sample Kolmogorov-Smirnov test showed that the intervals between LED and dot onset are not extracted from a standard normal distribution (Figure 9a,c,e), therefore a better indication for comparison between the temporal precision tests is the median (Figure 9b,d,f).

In the eye-detection scenario for the Tobii Pro SDK and the SRanipal, a median of 58.1 ms is found. In the gaze-contingent scenario, a median temporal precision of 58.1 ms is also found.

## 4. Discussion

New research using eye-tracking in VR has seen the emergence of more and more patient-friendly clinical applications intended to investigate and rehabilitate visual diseases. The current pilot study applied tailored data quality and temporal precision methods in VR to better understand how suitable is the manufacture’s information for future low vision usability as an online at-home virtual perimetry and enhancement implementation. As a show-case for healthy subjects, the methodology was applied to investigate the status-quo usability of the HTC VIVE Pro Eye. The results obtained opens new discussion relating to online eye-tracking usability in VR for novel at-home ophthalmology applications.

For an online virtual perimetry testing application, two different conditions were tested: head-still and head-free. The head-still was used to test the eye-tracking accuracy and precision data over a large visual field and at different HMD regions. For this purpose, fixational targets were fixed to the VR headset, and accuracy and precision were tested on a 2D plane covering ±26.6° of the visual field of the HMD both horizontally and vertically. The head-free scenario tested the effect of head movement over eye-tracking precision and data spillage. For this purpose, fixation was tested in a 3D environment while keeping the head stable and while moving. Both showed different limitations of the embedded eye-tracker.

The head-still condition evidenced that, in comparison to the manufacturer’s claim, spatial accuracy is worse than the reported values. Following previous VR eye-tracking accuracy research [36], eye-tracking data was more than three-time more inaccurate than the commercialized values with an average of 4.16°, SD: ±1.40°. Only the central target’s accuracy seems to be within the range of 0.5°–1.1° spatial accuracy reported by the manufacturer. The remaining targets have values outside the range and, as found in previous screen-based eye-tracking studies [31,37], the target position on the HMD screen affected eye-tracking data quality. Compared with the central line, at approximately 25° away from the midline, significant inaccuracy is found for the upper horizontal line and imprecision for the lower one.

The inaccuracy observed at the upper horizontal line indicated that fixations in regions above 25° from the midline are difficult. It is hypothesized that subjective facial configurations, such as the distance of the VR headset from the eyes, is shrinking the visual field and making fixation in that area more challenging. Very recent research has shown that the commercially reported visual field values of the most common VR headsets are the sum of monocular fields for each eye and the actual value that should be used to indicate the visual field extent is the monocular value [38]. It can be hypothesized, therefore, that the actual visual field measurement for the HTC Vive Pro Eye would be only half, ±27.5° horizontally and vertically. The eye-tracking methodology applied therefore tested accuracy at the edge of the VR headset’s possibilities in terms of the visual field, and this is reflected by the difficulty in fixating the extreme upper regions. Future studies using upcoming commercial HMDs with embedded eye-tracking should keep these restrictions in consideration.

As to the lower horizontal line, below 25° from the midline, eye-tracking data was found to be significantly more imprecise, and fixational points were more spread compared to the others. The spread of data points at the lowest edge of the HMD indicates effects due to reflections. It is known that reflections due to the surrounding environment, depending upon eye physiology, usage of corrective lenses, or due to the infrared camera position inside the VR headset, can lead to errors in the eye-tracking data [39]. The observed changes in data quality across the population indeed point towards external factors affecting eye-tracking data quality. Therefore, it is hypothesized that the observed deviations could be affected by all three above mentioned effects. Traditional calibration methodology can correct for each user’s eye physiological characteristics [40]. Nonetheless, it can still be challenged by light conditions, the eye-correction used, reflections, and head movement [28] and this is what was found in our preliminary study as well.

Novel calibration methodology suitable in moving scenarios for online eye-tracking usage in VR could be used instead [41]. More and more research is being done in that direction. The most promising, which could overcome most of the current study’s challenges, uses smooth pursuit to self-calibrate the system while a task is still being performed in VR. Results have shown that smooth pursuit calibration can overcome challenges such as differences in eye physiology, head movement, and problems in keeping a stable fixation [42,43] which are common in patients with visual loss. For a visual grasping mode, instead of using stimuli that change in brightness, limiting eye-tracking data [28], moving stimuli could be used to attract a patient’s attention towards a new test area, which can occur in concomitance with self-calibration. Smooth-pursuit tasks in combination with head movements do not influence patients with both binocular and monocular visual loss more than normally sighted participants [44]. Hence, self-calibration systems that use smooth-pursuit for online visual field perimetry testing could overcome problems due to light conditions, patient fixational stability, eye physiology, and head movements. In future studies, a self-calibrating smooth pursuit could be applied both to normal and other patients, and the result could be compared to the current data.

The head-free condition evidenced how precision and data loss can be influenced by head movement: precision is lowest, and a double amount of data loss occurs while moving. The results obtained are pertinent with head-mounted eye tracker studies [28,35,45].

From the data quality analyzed, it can be concluded that the feasibility of the HTC Vive Pro Eye as an online objective visual grasp tool that could detect the early onset of glaucoma at eccentricities above ± 25° [46] is very restricted. With high inaccuracy and imprecision above ± 25° from the midline, and eye-tracking imprecision and data spillage during movement, its status-quo usage in online visual field testing is limited. The manufacturer’s information shows no indication of these restrictions; therefore, the current pilot study provided additional eye-tracking data information for visual field online low-vision applications.

As to its application for online visual enhancement clinical studies that require a limited temporal precision and lack reliable and direct temporal precision measurements, additional conditions should be kept in mind. The display refresh rate can make a difference between a good or an acceptable latency level [20,47]. The eye tracker used in the HTC Vive Pro Eye has a higher refresh rate than the display, therefore, for this system, one part of the latency’s variance can be due to the display’s refresh. Additionally, ideally the display should be updated immediately at the end of each saccade. This is limited in practice since a lag always exists between the identification of saccade ending, rendering the new image, transmitting it, and displaying it, due to hardware differences [48]. For example, rendering the image can take from 25 to 150 ms [49,50,51] and an acceptable level of the system’s latency depends on the application.

The new objective and automatic temporal precision tests showed that there is no difference between the detection of an eye and a gaze-contingent scenario. Furthermore, displaying data through the Tobii Pro SDK or the SRanipal SDK makes no difference in terms of temporal precision. For all the tests conducted, the median is a good indicator of temporal precision. The value of 58.1 ms makes the system suitable for patient-friendly visual enhancement applications. Indeed, it has been discussed that for changes in the peripheral areas of vision, latencies between 50 and 70 ms are well accepted because visual loss simulations are applied in the periphery, and they are not usually detected [48,52]. This happens because changes in the post saccade area mostly overlap with changes in the pre-saccadic [47]. If a saccade has a maximum duration of 54 ms and peripheral changes can go undetected up to 70 ms, the HTC Vive Pro Eye’s eye-tracker is suitable as a responsive and undetectable online visual enhancement software.

## 5. Conclusions

In this study, the goal was to assess the preliminary eye-tracking status-quo capabilities of the HTC VIVE Pro Eye in a pilot number of healthy subjects to test its potentiality for future online clinical low-vision applications. Preliminary results indicate that the status-quo of eye-tracking embedded in the HTC VIVE Pro Eye has limitations for online VR perimetry testing and is generally suited as a low vision enhancement software. The results obtained added essential discussion points to be considered for future and upcoming VR headsets that want to use embedded eye-tracking as a virtual perimetry testing. The correctness of the actual reported visual field expansion of the VR headset and its relation to eye-tracking data need to be considered and additionally tested over a more heterogeneous subject population. Furthermore, a more suited smooth pursuit online self-calibration system could be considered for ongoing VR perimetry when considering using VR headsets for patients.

## Figures and Tables

**Table 1:** Mean angle accuracy and RMS precision for the 25 different targets across the visual field (VF) tested for left (L), right (R), and left-right average (B).

VF (°)	−27°			−13°			0°			13°			27°		
	L	R	B	L	R	B	L	R	B	L	R	B	L	R	B
27°	10.77	10.09	9.95	5.84	5.16	5.28	3.05	3.63	3.1	5.13	4.53	4.58	8.01	8.6	7.93
	2.64	2.67	2.29	2.27	1.91	1.86	2.84	4.29	2.79	1.9	1.98	1.72	3.18	2.18	2.25
13°	4.21	3.66	3.61	3.8	3.47	3.57	2.87	3.19	2.96	3.28	3.67	3.36	3.08	4.82	3.55
	2.16	2.4	1.94	2.31	2.11	1.87	1.87	2.1	1.67	2.12	2.06	1.75	2.22	2.03	1.88
0°	2.4	2.66	2.2	3.09	3.3	3.11	0.94	1.02	0.74	3.12	2.98	2.87	2.34	3.21	2.37
	1.95	2.08	1.79	1.73	1.81	1.55	1.64	1.8	1.45	1.98	1.95	1.68	1.94	1.95	1.76
−13°	4.05	5.32	4.3	4.14	4.16	4.1	3.27	2.34	2.73	4.78	3.98	4.33	3.49	4.98	3.75
	2.02	3.4	2.35	1.63	3.16	2.03	1.73	3.91	2.31	1.87	2.08	1.67	2.09	2.35	1.92
−27°	6.95	9.44	7.41	4.57	6.68	5.39	2.44	7.13	4.06	4.63	8.12	6.01	5.62	8.7	6.44
	2.02	4.81	2.89	2.51	4.32	2.81	3.12	7.13	4.06	2.87	5.99	3.5	4.13	3.4	2.95

**Table 2:** Mean accuracy and RMS precision across different percentiles of each target in the head-still condition.

Percentile (Head-Still)	Accuracy (°)	Precision (°)
25%	3.21	1.63
50%	3.98	1.95
75%	4.88	2.51
90%	6.06	3.55

## Data Availability

Data is available in figshare under HTCProEyeDataQuality&TemporalPrecision_Data. Link: https://figshare.com/s/dee0b7285b98748b512e, accessed on 2 February 2021.
